# Niclosamide inhibits leaf blight caused by *Xanthomonas oryzae* in rice

**DOI:** 10.1038/srep21209

**Published:** 2016-02-16

**Authors:** Sung-Il Kim, Jong Tae Song, Jin-Yong Jeong, Hak Soo Seo

**Affiliations:** 1 Department of Plant Science and Research Institute of Agriculture and Life Sciences, Seoul National University, Seoul 151-921, Korea; 2 School of Applied Biosciences, Kyungpook National University, Daegu 702-701, Korea; 3 Department of Convergence Medicine and Asan Institute for Life Sciences, Asan Medical Center, University of Ulsan College of Medicine, Seoul 05505, Republic of Korea; 4 Plant Genomics and Breeding Institute, Seoul National University, Seoul 151-921, Korea; 5 Bio-MAX Institute, Seoul National University, Seoul 151-818, Korea

## Abstract

Rice leaf blight, which is caused by the bacterial pathogen *Xanthomonas oryzae* pv*. oryzae* (*Xoo*), results in huge losses in grain yield. Here, we show that *Xoo*-induced rice leaf blight is effectively controlled by niclosamide, an oral antihelminthic drug and molluscicide, which also functions as an anti-tumor agent. Niclosamide directly inhibited the growth of the three *Xoo* strains PXO99, 10208 and K3a. Niclosamide moved long distances from the site of local application to distant rice tissues. Niclosamide also increased the levels of salicylate and induced the expression of defense-related genes such as *OsPR1* and *OsWRKY45*, which suppressed *Xoo*-induced leaf wilting. Niclosamide had no detrimental effects on vegetative/reproductive growth and yield. These combined results indicate that niclosamide can be used to block bacterial leaf blight in rice with no negative side effects.

Bacterial blight is one of the most serious diseases of rice and is especially prevalent in irrigated and rain-fed lowland areas. Bacterial blight has the following three symptoms: leaf blight, kresek (seedling blight or wilt phase of the syndrome), and pale-yellow leaves^1^. This disease is sometimes referred to as bacterial leaf blight, indicating that the leaf blight phase of the syndrome is the most distinct and commonly observed phase^1^. The most common symptom of this disease is leaf wilting, especially in young leaves, because the bacterial blight pathogen multiplies in xylem elements and primarily invades the vascular tissue^2^.

Bacterial blight in rice is caused by *Xanthomonas oryzae* pv*. oryzae* (*Xoo*)^3^ and results in huge losses in grain yield. *Xoo* is a phytopathogenic Gram-negative bacterium that belongs to the family *Pseudomonadaceae. Xoo* enters the plant through wounds or hydathodes, multiplies in the epitheme, moves to the xylem vessels, and undergoes active multiplication, which results in blight disease symptoms in rice leaves. *Xoo* produces a range of virulence factors, including exopolysaccharides, extracellular enzymes, iron-chelating siderophores, and type III secretion-dependent effectors, which are collectively essential for virulence^4^,^5^,^6^,^7^,^8^.

Numerous studies have investigated rice protection from bacterial blight. Three different strategies, biological control, chemical control, and genetic resistance, have been employed to manage plant diseases such as bacterial leaf blight. Specific bacterial strains have been used for biological control of rice leaf blight; for example, fresh suspensions and powdered formulations of plant growth-promoting rhizobacteria^9^. *Bacillus* spp. and *Pseudomonas* spp. also have been used to suppress rice leaf blight^10^. However, pathogen variation and the lack of suitable biological agents limit the efficacy of biological controls, although such measures remain a suitable method for disease control and management because they are environmentally friendly.

Numerous chemicals have been tested for chemical control of bacterial leaf blight. For example, leaf blight lesions in rice were reduced by application of bleaching powder containing 30% chlorine^11^. The broad-spectrum antibiotics benzylpenicillin, ampicillin, kanamycin, streptomycin, chloramphenicol, and sinobionic also inhibit the growth of *Xoo* isolates, although their effects were examined only *in vitro*^12^. Benzothiadiazole, a known plant activator, exerts protective effects against a broad spectrum of diseases including bacterial leaf blight, without inducing major adverse effects on plant growth when applied at appropriate dosages^13^. Probenazole also protects rice from blast caused by the fungal pathogen *Magnaporthe grisea*^14^. Streptomycin has been used for crop protection from bacterial and fungal diseases, including fire blight, bacterial blight, and soft rot^15^. Oxytetracycline, gentamycin, and oxolinic acid have been used to control several bacterial and fungal diseases^15^. However, an effective and economical chemical treatment for rice leaf blight has not been established, although research and development are ongoing.

Enhancing plant genetic resistance is an effective method for controlling bacterial leaf blight disease. A number of studies have identified plant genes that confer resistance against *Xanthomonas* bacteria. At least 38 bacterial leaf blight resistance gene (*R* genes), designated in series from *Xa1* to *Xa38*, have been identified^16^,^17^. Six of these genes have been cloned (*Xa1*, *xa5*, *xa13*, *Xa21*, *Xa3*/*Xa26*, and *Xa27*), and six additional genes have been physically mapped (*Xa2*, *Xa4*, *Xa7*, *Xa30*, *Xa33*, and *Xa38*)^16^,^17^,^18^,^19^,^20^,^21^,^22^. Two major classes of *R* genes, receptor kinase (RLK) and nucleotide-binding site leucine rice repeat (NBS)-LRR, are involved in disease resistance in rice. *Xa21* is the first *R* gene of the RLK class to be cloned with a broad spectrum of resistance, and *Xa1* is an *R* gene of the NBS-LRR class^23^, which is the largest *R* gene class conferring resistance against bacteria, fungi, and viruses^24^. Other *R* genes, including *xa5* and *xa13*, encode proteins such as a small subunit of the transcription factor IIA (TFIIAγ) and a plasma membrane protein, respectively^25^,^26^. These data have been used to develop a breeding program for bacterial leaf blight-resistant rice. The introduction of resistance genes *xa5*, *xa13*, and *Xa21* into new cultivars conferred a high level of resistance against *Xoo*^27^, indicating that germplasm screening against bacterial leaf blight and breeding-mediated transfer of resistant genes to target cultivars can generate new rice cultivars with increased resistance to bacterial leaf blight disease. Large-scale experiments, including microarrays, were used to isolate genes related to bacterial leaf blight; a large number of candidate genes was selected, although their functions were not clearly identified^28^,^29^. A transgenic approach was employed to protect rice from bacterial leaf blight and fungal blast. For example, transgenic rice overexpressing cecropin B (an antibacterial peptide from *Bombyx mori*) showed increased resistance to bacterial leaf blight^30^. The introduction of multiple bacterial blight resistance genes (*Xa4*, *xa5*, *xa13*, and *Xa21*) into rice conferred resistance to six *Xoo* bacteria^31^. Rice transformants overexpressing WRKY30 are resistant to *Xoo*^32^ and the fungal pathogen *Magnaporthe oryzae*^33^, and those overexpressing WRKY45 displayed markedly enhanced resistance to bacterial leaf blight and fungal blast disease^34^,^35^,^36^. These results suggest that transgenic approaches can be effectively utilized to develop disease-resistant rice.

Although several biological, chemical, and genetic control approaches have yielded improvements in rice protection from bacterial leaf blight, there was still a need for an effective approach that would provide large-scale protection. Therefore, we focused our current efforts on screening known chemicals for their effects on rice leaf blight. We are interested in the discovery of a master regulator that effectively functions to protect animals and plants from infectious diseases mediated by bacteria, fungi, and/or parasites. Therefore, we first considered infectious diseases of humans. We selected two drugs that cure infectious diseases in humans, auranofin [3,4,5-triacetyloxy-6-(acetyloxymethyl)oxane-2-thiolate] and niclosamide [5-chloro-*N*-(2-chloro-4-nitrophenyl)-2-hydroxybenzamide], for further analysis. However, because auranofin is much more expensive than niclosamide, we focused our initial screen on niclosamide. Niclosamide has been widely used since 1960 to treat gastrointestinal tapeworm infections in both humans and animals. Recent studies show that niclosamide has antiviral activity against the severe acute respiratory syndrome virus^37^, anti-anthrax toxin properties^38^, and anti-neoplastic activity^39^. Niclosamide strongly induces LC3-positive autophagosomes^40^, inhibits the Wnt/Frizzled pathway^41^, suppresses the autonomous notch-signaling pathway^42^, and inhibits mTOR signaling^43^. Niclosamide uncouples mitochondrial oxidative phosphorylation^44^ and thereby slows cell growth. These combined results indicate that niclosamide has various curative effects on humans and animals. We reasoned that niclosamide might affect plant disease responses by modulating disease signaling pathways as a broad spectrum regulator. Therefore, we investigated the effect of niclosamide on bacterial leaf blight in rice.

Here, we show that niclosamide blocks rice leaf wilting mediated by *Xoo* bacteria, both locally and systemically, without negatively affecting plant growth. The results suggest that niclosamide may be used to prevent rice leaf blight caused by bacterial pathogen attack.

## Results

### Niclosamide inhibits *Xoo* bacterial growth

**Figure 1:**
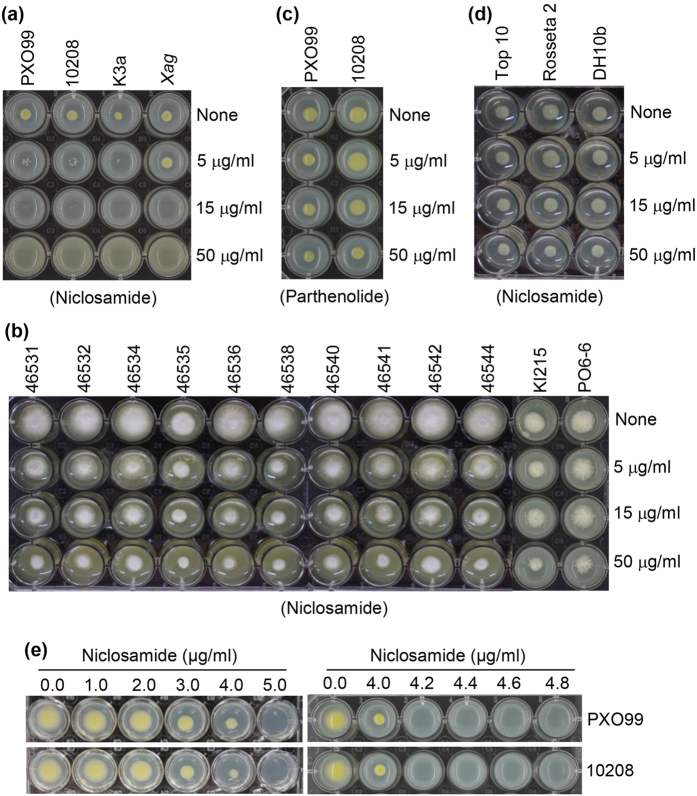
Effects of niclosamide on the growth of bacterial and fungal pathogens. (**a**) *Xoo* strains PXO99, 10208, and K3a, and a *Xag* strain were grown on PSA medium containing different niclosamide concentrations. (**b**) Growth inhibitory effect of niclosamide on twelve *M. oryzae* strains: 46531, 46532, 46534, 46535, 46536, 46538, 46540, 46541, 46542, 46544, KI215, and PO6-6. (**c**) Effects of parthenolide against strains PXO99 and 10208, which were used as controls. (**d**) Effects of niclosamide on the growth of three different Gram-negative *E. coli* strains: Top10, Rosseta2, and DH10b. (**e**) Minimum inhibitory concentration (MIC) of niclosamide against *Xoo* strains PXO99 and 10208.

To evaluate the functional effect of niclosamide on bacterial blight, we first examined its effect on the growth of *Xoo* bacteria using three different strains, PXO99, 10208, and K3a, and *Xanthomonas axonopodis* pv. *glycines* (*Xag*). The results showed that growth of the *Xoo* strains was completely inhibited by 5 μg/ml niclosamide, whereas *Xag* growth was inhibited by 15 μg/ml niclosamide (Fig. 1a). *Xag* is a pathogen that causes bacterial leaf pustule disease in soybean^45^. We also examined the effect of niclosamide on the growth of the fungal pathogen *Magnaporthe oryzae*. Niclosamide did exert an inhibitory effect on the growth of 12 *M. oryzae* strains, although the inhibition was considerably weaker than that against *Xoo* (Fig. 1b).

We also tested the effects of parthenolide, a sesquiterpene lactone with anti-tumor activity, on the growth of the *Xoo* strains PXO99 and 10208. Parthenolide failed to inhibit the growth of either *Xoo* strain at a concentration of 5 μg/ml, although it slightly inhibited bacterial growth at 50 μg/ml (Fig. 1c). We also tested the effect of niclosamide on the growth of three *E. coli* strains, Top10, Rosseta2, and DH10b, which served as Gram-negative control bacteria. At lower concentrations, niclosamide had no effect on the growth of these *E. coli* strains, although it slightly inhibited growth at 50 μg/ml (Fig. 1d).

To determine the minimum inhibitory concentration (MIC) of niclosamide on PXO99 and 10208 growth, we tested concentrations ranging from 0–5 μg/ml. Both *Xoo* strains could grow in the presence of 4 μg/ml niclosamide, but not 5 μg/ml niclosamide (Fig. 1e; left panel). We further narrowed the MIC of niclosamide from 4–5 μg/ml. As shown in Fig. 1e, the growth of both PXO99 and 10208 was completely inhibited by 4.2 μg/ml niclosamide (Fig. 1e; right panel), indicating that 4.2 μg/ml niclosamide is the MIC for both *Xoo* strains.

### Rice bacterial blight is blocked by niclosamide

We examined whether niclosamide blocks bacterial blight in rice. For this experiment, we grew the rice cultivar Nipponbare in a growth chamber for 12 weeks (before bolting), and subjected the plants to pathogen and niclosamide treatment.

**Figure 2:**
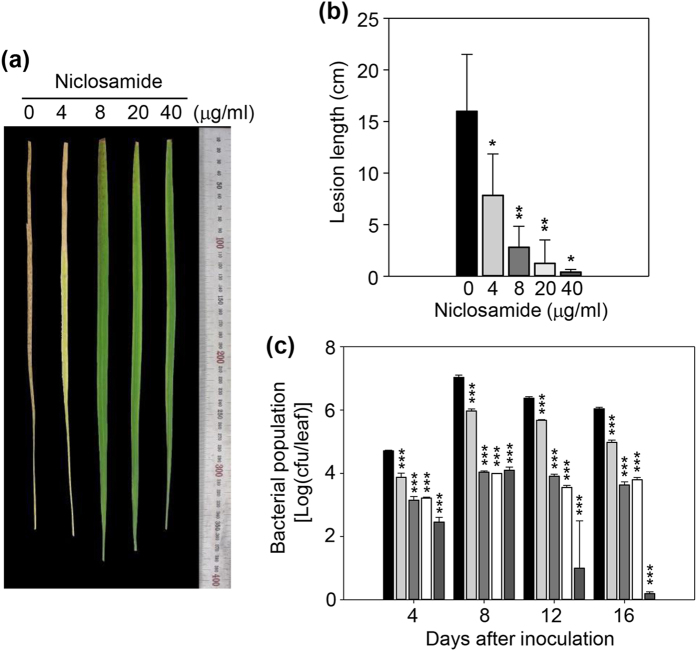
Niclosamide dosage effects on rice disease responses to the representative *Xoo* strain PXO99. (**a**) Leaves were inoculated with PXO99 bacterial suspension using the leaf-clipping method and the plants sprayed with different niclosamide concentrations. Photographs were taken at 16 days post-inoculation. (**b**) Lesion development was examined by measuring lesion length in PXO99-treated plant leaves or niclosamide- and PXO99-treated plant leaves. Standard deviations of the means are indicated by vertical bars. Asterisks indicate statistically significant differences in lesion lengths (*p < 0.01; **p < 0.001; Student’s *t*-test) between untreated and niclosamide-treated leaves. (**c**) Leaves of PXO99-treated plants or niclosamide- and PXO99-treated plants were sampled at the indicated time points. The samples were ground, suspended in sterile water, and plated onto peptone-sucrose agar medium containing cephalexin. The numbers of bacterial colonies were then counted. Standard deviations of the means are indicated by vertical bars. 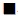, PXO99 only; 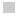, PXO99 plus 4 μg/ml niclosamide; 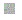, PXO99 plus 8 μg/ml niclosamide; □, PXO99 plus 20 μg/ml niclosamide; 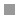, PXO99 plus 40 μg/ml niclosamide. Standard deviations of the means are indicated by vertical bars. Asterisks indicate statistically significant differences in bacterial populations (***p < 0.001; Student’s *t*-test) between untreated and niclosamide-treated leaves at different time points after PXO99 inoculation.

First, to determine the minimum concentration of niclosamide that would block bacterial blight, we examined the niclosamide dosage effect on disease responses to the representative *Xoo* strain PXO99. We inoculated PXO99 onto Nipponbare using the leaf-clipping method, sprayed the plants with different niclosamide concentrations, and examined the phenotypes of plants treated with pathogen only or pathogen plus niclosamide. Leaf wilting did not develop in plants treated with ≥8 μg/ml niclosamide (Fig. 2a). We also estimated lesion development by measuring lesion length. Lesion development also was significantly inhibited by 8 μg/ml niclosamide, although a small lesion was still detected at this concentration (Fig. 2b). Lesion development gradually declined with increasing niclosamide concentrations (Fig. 2b). We also examined the levels of bacterial growth, and found that population levels of *Xoo* bacteria declined with increasing niclosamide concentrations (Fig. 2c).

**Figure 3:**
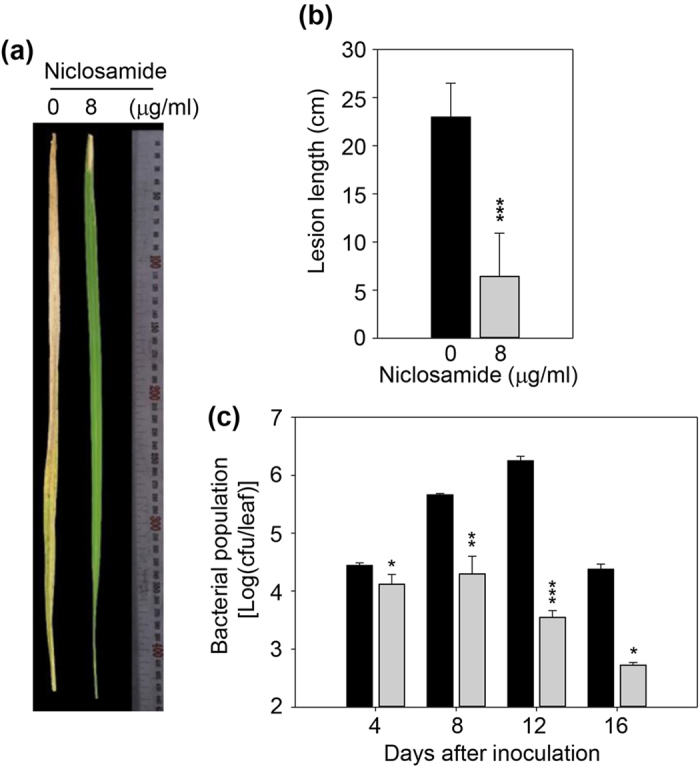
Niclosamide effects on rice disease responses to the representative *Xoo* strain PXO99. (**a**) Leaves were inoculated with bacterial suspension using the leaf clipping method and sprayed with 8 μg/ml niclosamide. (**b**) Lesion development was examined by measuring lesion length using the leaves of PXO99-treated plants or niclosamide plus PXO99-treated plants. The standard deviations of the means are indicated by vertical bars. Asterisks indicate statistically significant differences in lesion lengths (***p < 0.001; Student’s *t*-test) between untreated and niclosamide-treated leaves. (**c**) Leaves of PXO99-treated plants or niclosamide- and PXO99-treated plants were sampled at the indicated time points. Samples were ground, suspended in sterile water, and plated onto peptone-sucrose agar medium containing cephalexin. The numbers of bacterial colonies were then counted. Standard deviations of the means are indicated by vertical bars. 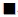, PXO99 only; 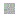, PXO99 plus niclosamide. Standard deviations of the means are indicated by vertical bars. Asterisks indicate statistically significant differences in bacterial populations (*p < 0.01; **p < 0.001; ***p < 0.001; Student’s *t*-test) between untreated and niclosamide-treated leaves at different time points after PXO99 inoculation.

We next examined the effect of niclosamide on rice disease responses to *Xoo* bacteria after treatment with PXO99. First, Nipponbare rice was inoculated with PXO99 by the leaf-clipping method. After incubation for four days until leaf blight lesions with a length of 2 cm developed, the plants were sprayed with 8 μg/ml niclosamide (Fig. 3). Leaf blight was completely blocked in niclosamide-treated leaves (Fig. 3a). Lesion development also was completely blocked in niclosamide-treated leaves (Fig. 3b), and PXO99 growth was inhibited (Fig. 3c).

### Niclosamide has a systemic effect on rice disease response

**Figure 4:**
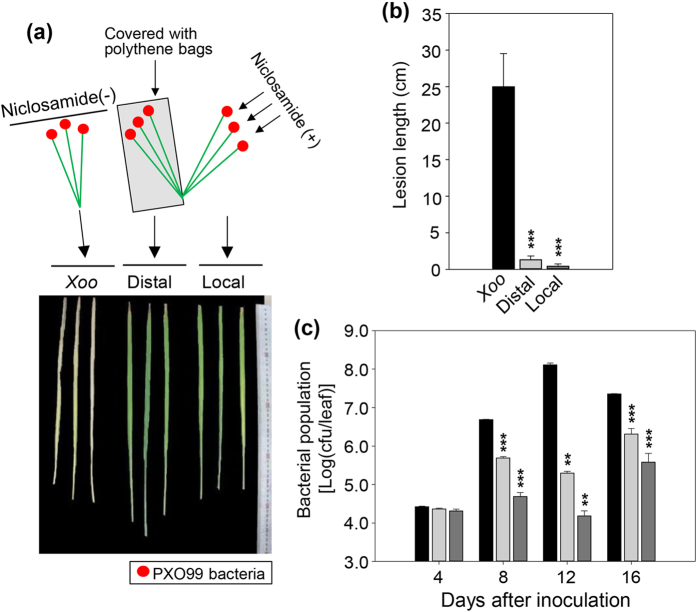
Systemic effects of niclosamide on rice disease responses to the representative *Xoo* strain PXO99. (**a**) Leaves were inoculated with PXO99 bacterial suspension using the leaf-clipping method (left panel). Half of the leaves were covered with polythene bags (middle panel) and the remaining leaves were sprayed with niclosamide (right panel). Photographs were taken at 16 days post-inoculation. (**b**) Lesion development was examined by measuring lesion length in PXO99-treated plant leaves or the local and distal leaves of niclosamide- and PXO99-treated plant leaves. Standard deviations of the means are indicated by vertical bars. Asterisks indicate statistically significant differences in lesion lengths (***p < 0.001; Student’s *t*-test) between untreated and niclosamide-treated local leaves or between untreated and niclosamide-treated systemic leaves. (**c**) Leaves of PXO99-treated plants or local and distal leaves of niclosamide- and PXO99-treated plants were sampled at the indicated time points. Samples were ground, suspended in sterile water, and plated onto peptone-sucrose agar medium containing cephalexin. The numbers of bacterial colonies were then counted. Standard deviations of the means are indicated by vertical bars. 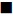, PXO99 only; 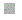, PXO99 plus niclosamide (distal); 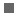, PXO99 plus niclosamide (local). Standard deviations of the means are indicated by vertical bars. Asterisks indicate statistically significant differences in bacterial populations (**p < 0.001; ***p < 0.001; Student’s *t*-test) between niclosamide-treated local and systemic leaves at different time points after PXO99 inoculation.

We next investigated whether niclosamide could move from the site of local application to distant tissues and subsequently inhibit PXO99-mediated leaf wilting. We inoculated half of the leaves of Nipponbare plants with PXO99 and covered them with polythene bags. Then, we sprayed the non-inoculated leaves with niclosamide and examined leaf blight in both local and systemic leaves. Leaf wilting and lesion development were completely inhibited in PXO99-inoculated leaves that had not been treated with niclosamide (Fig. 4a,b). PXO99 growth also was significantly inhibited in the inoculated leaves (Fig. 4c).

**Figure 5:**
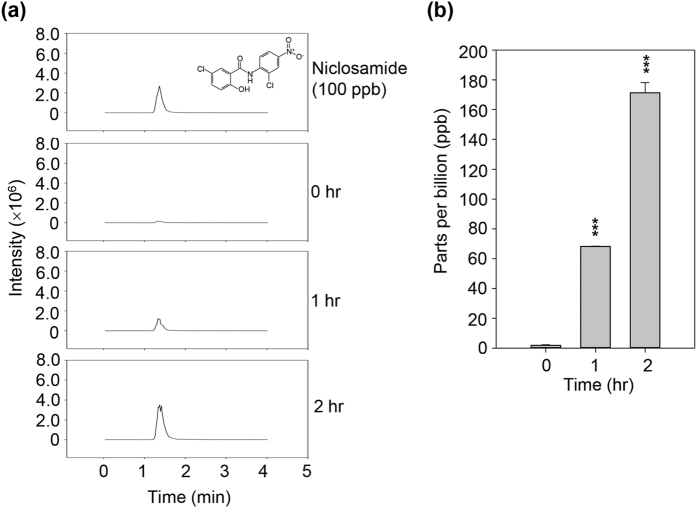
Niclosamide detection in distal leaves. Leaves of 80-day-old plants were sprayed with 8 μg/ml niclosamide and compared with untreated distal leaves sampled at the indicated time points. Samples were extracted with methanol, and niclosamide levels in the extracts were detected by HPLC. (**a**) HPLC chromatogram. Inset indicates the chemical structure of niclosamide. (**b**) Niclosamide levels at each time point were expressed in graph form. Asterisks indicate statistically significant differences in niclosamide amounts (***p < 0.0001; Student’s t-test) between untreated local leaves and niclosamide-treated systemic leaves.

Next, we examined the long-distance movement of niclosamide by extracting niclosamide from niclosamide-treated leaves and untreated systemic leaves. Niclosamide levels gradually increased in systemic leaves (Fig. 5a,b), indicating that niclosamide can systemically move from niclosamide-treated local leaves to untreated distal leaves.

### Niclosamide induces the expression of defense-related genes

**Figure 6:**
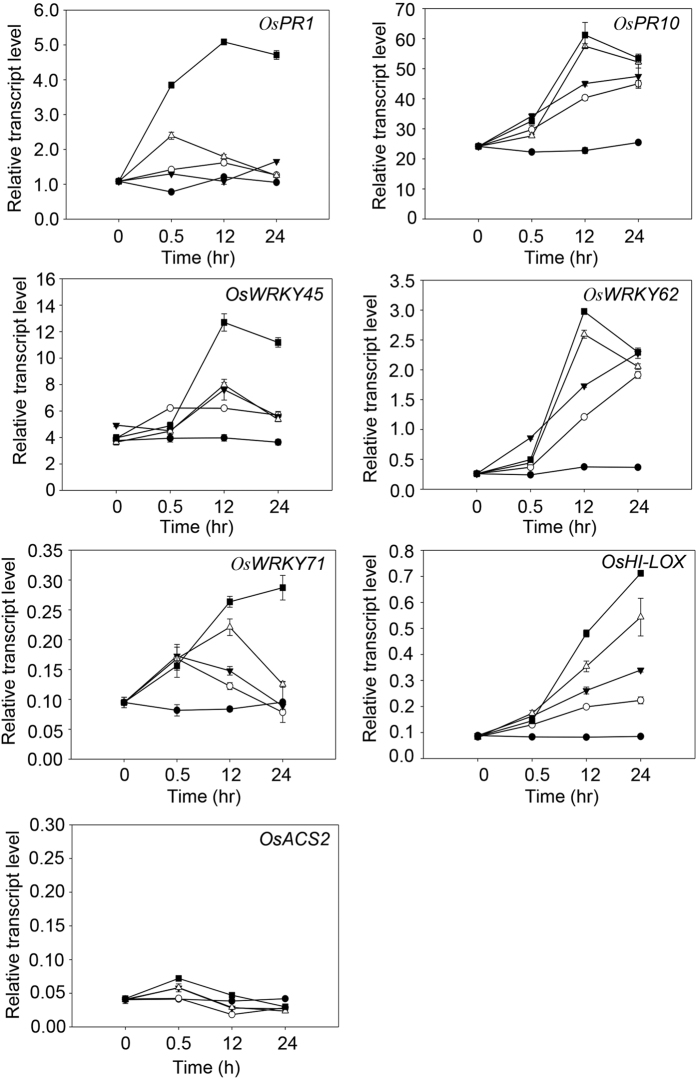
Expression analysis of pathogen-related genes in niclosamide-treated leaves. RNA samples were extracted from leaves treated with mock (●), 4 μg/ml niclosamide (○), 8 μg/ml niclosamide (▼), 20 μg/ml niclosamide (Δ), and 40 μg/ml niclosamide (■) at four time points (0, 0.5, 12, and 24 h). *OsPR1*, *OsPR10*, *OsWRKY45*, *OsWRKY62*, *OsWRKY71*, *OsHI-LOX*, and *OsACS2* transcripts were amplified by qRT-PCR using gene-specific primers.

We next examined whether niclosamide protects PXO99-infected plants through the induction of pathogen-related gene expression. We treated the rice cultivar Nipponbare with different niclosamide concentrations for 24 h, and then extracted total RNA from niclosamide-treated and untreated control plants. We performed qRT-PCR analysis to examine the transcript levels of seven defense-related genes, including *OsPR1*, *OsPR10*, *OsWRKY45*, *OsWRKY62*, *OsWRKY71*, *OsHI-LOX*, and *OsACS2*. The results showed that *OsPR1*, *OsPR10*, *OsWRKY45*, *OsWRKY62*, *OsWRKY71*, and *OsHI-LOX* were induced by niclosamide treatment, and that transcript expression levels increased with increasing niclosamide levels (Fig. 6). However, *OsACS2* expression was only slightly increased 30 min after treatment with 40 μg/ml niclosamide, which suggests that its expression was not affected by niclosamide (Fig. 6).

**Figure 7:**
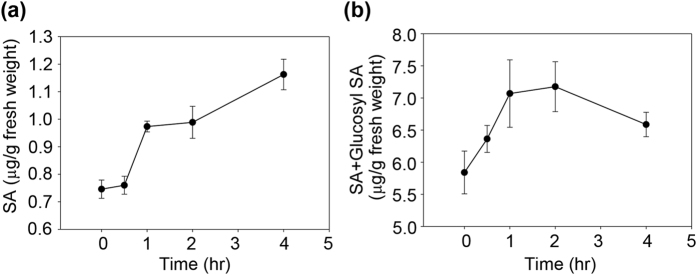
Salicylic acid content in niclosamide-treated rice. Levels of free SA and glucosyl-SA were evaluated in leaves of rice plants treated with 8 μg/ml niclosamide. Samples were harvested and SA and glucosyl-SA were extracted using a methanol-based method. Free SA (**a**) or total SA (**b**) content combined with free SA and glucosyl-SA were analyzed by HPLC. Results are expressed as the means ± S.D. (*n* = 3).

The pathogenesis-related genes *OsPR1*, *OsPR10*, *OsWRKY45*, *OsWRKY62*, and *OsWRKY71* are induced by high salicylate (SA) levels^46^,^47^,^48^,^49^; therefore, we measured free SA and glucosyl-SA levels in the leaves of niclosamide-treated rice plants. SA levels increased in response to niclosamide treatment (Fig. 7a,b), indicating that niclosamide induces the expression of pathogenesis-related genes by increasing the levels of SA and SA-conjugates.

### Niclosamide has no effect on rice growth and development

**Figure 8:**
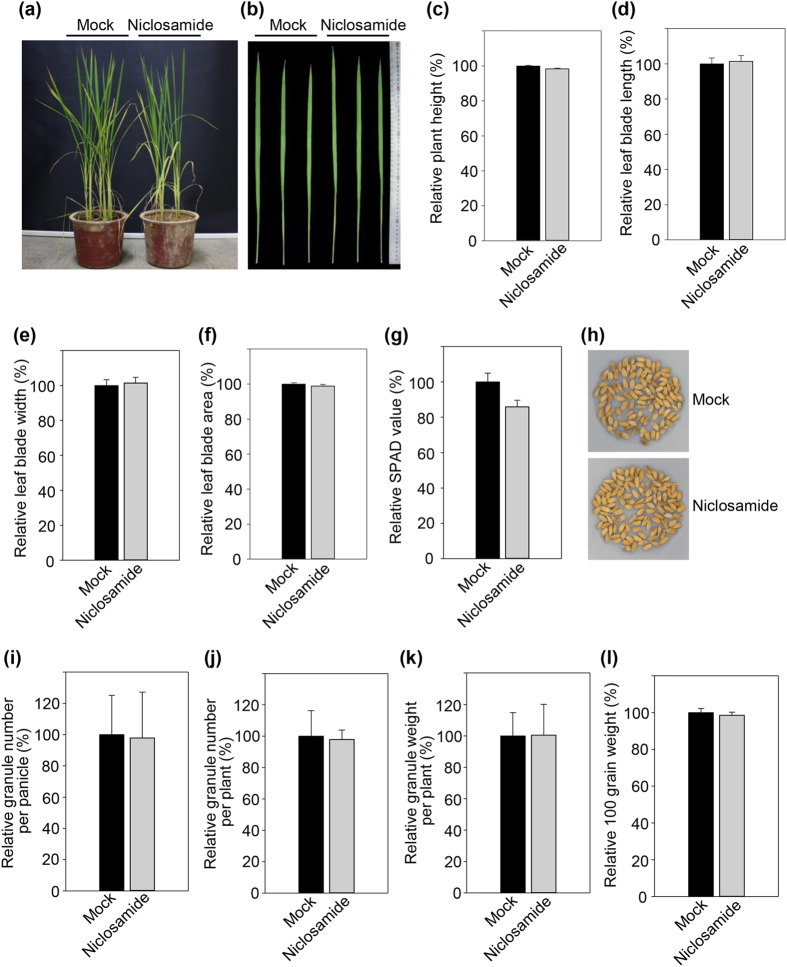
Effect of niclosamide on rice growth and development. (**a**) Three-week-old plants were sprayed with 8 μg/ml niclosamide every 4 days. After 70 days, the plants were photographed. (**b**) Photographs of leaves from untreated and niclosamide-treated plants. (**c**–**f**) Height, leaf blade length and width, and leaf blade area of leaves from untreated and niclosamide-treated plants were measured. (**g**) SPAD values were examined in leaves of untreated and niclosamide-treated plants. (**h**–**l**) Granule phenotype, number, and weight were evaluated for mature seeds of untreated and niclosamide-treated plants.

We examined the effect of niclosamide on rice growth and development from the vegetative stage to seed maturation. Three-week-old rice plants were treated with 8 μg/ml niclosamide at 4-day intervals and the phenotypic characteristics of niclosamide-treated and -untreated plants were examined at 80 days after planting. Plant height and leaf characteristics were not altered by niclosamide treatment, although the contents of chlorophyll and other compounds (SPAD values) were slightly reduced in niclosamide-treated plants (Fig. 8a–g). Seed characteristics such as color, number, and weight were not affected by niclosamide treatment (Fig. 8h–l), indicating that niclosamide does not have any detrimental effects on rice growth, development, and grain yield.

## Discussion

*Xoo*-mediated leaf blight is one of the most devastating rice diseases worldwide. For the past two decades, substantial efforts have been made to identify and isolate bacterial blight-resistance genes. In this study, we used an alternative chemical approach to protect rice from *Xoo* infection.

More than 30 drugs, including antibiotics, have been utilized to protect crops from pathogen attack. Some of these have broad-spectrum bactericidal and fungicidal activity, whereas others specifically target bacteria or fungi. Oxytetracycline and streptomycin are commonly used antibiotics in humans and plants^15^, suggesting that some types of human drugs can positively control diseases in plants. Therefore, we screened human drugs for their ability to prevent rice leaf blight disease. Our long-term goal is to identify a master regulator that inhibits pathogenic disease in both plants and humans.

Niclosamide was initially characterized as an oral antihelminthic drug and molluscicide^36^. Several recent studies report that niclosamide is active against cancer cells^39^,^50^,^51^, which indicates that niclosamide has broad-spectrum disease control activity. Therefore, we examined whether niclosamide had inhibitory activity against rice leaf blight, and found that the leaf blight symptoms were suppressed by niclosamide through inhibition of *Xoo* growth and lesion development (Figs 2 and 3). Niclosamide moved systemically from the local application site to *Xoo*-inoculated distal leaves, and inhibited lesion development and leaf wilting by inhibiting *Xoo* growth in distal leaves (Fig. 4). We examined the effect of niclosamide on the growth of three *Xoo* strains, and found that it inhibited their growth (Fig. 1a,e). Next, we evaluated whether niclosamide induced the expression of the defense-related genes *OsPR1*, *OsPR10*, *OsWRKY45*, *OsWRKY62*, *OsWRKY71*, and *OsHI-LOX*, and detected higher transcript levels for these genes (Fig. 6). These results clearly indicate that niclosamide blocks the development of rice leaf blight by directly inhibiting *Xoo* bacterial growth (Figs 2c and 3c) and/or by inducing defense-related gene expression (Fig. 6). Niclosamide also inhibited *Xag* growth, which causes bacterial leaf pustule disease in soybean^45^, although its inhibitory effect against *Xag* was weaker than that against *Xoo* strains (Fig. 1a). This suggests that niclosamide may inhibit the growth of select bacterial pathogens of other crops and thereby protect them from disease.

Jasmonate (JA) and SA function in plant defense pathways by inducing the expression of numerous genes^52^. Treatment of rice leaves with benzothiadiazole or probenazole induced SA accumulation and increased resistance to bacterial blight and fungal blast caused by *Xoo* and *M. grisea*, respectively^13^,^14^,^34^. Our qRT-PCR analysis showed that niclosamide induced the expression of SA-dependent genes in rice leaves (Fig. 6). We investigated whether niclosamide induced defense mechanisms directly by functioning like a phytohormone, or indirectly via SA-mediated signaling pathways. We measured the levels of free SA and its conjugate glucosyl-SA, and found that their levels were higher in niclosamide-treated leaves than in control leaves (Fig. 7). This indicates that increase in SA level can contribute to the blockage of *Xoo* lesion development by niclosamide.

WRKY proteins are involved in JA response pathways in rice. OsWRKY30 triggers the expression of JA-responsive genes and JA accumulation, and provides rice with resistance to fungal pathogens *Rhizoctonia solani* and *M. grisea*^33^. *OsWRKY45* expression regulates JA accumulation and resistance to the rice blast fungus *M. grisea*^53^. Our results showed that the expression of *OsHI-LOX*, a JA-responsive gene, was induced in niclosamide-treated leaves (Fig. 6). This indicates that JA-dependent defense signaling pathways can also contribute to the blockage of *Xoo* lesion development by niclosamide.

Pathogens are sensitive to the activities of certain classes of drugs. The antibiotic streptomycin, which is commonly used in animals and humans, protects crops from some bacterial and fungal pathogens^15^. Niclosamide serves as an oral antihelminthic drug, molluscicide, and anti-tumor agent in human disease; therefore, we reasoned that niclosamide might have beneficial effects on bacterial and fungal diseases of rice. Niclosamide inhibited growth of the fungal pathogen *M. oryzae*, although this effect was much weaker than that against pathogenic *Xoo* bacteria (Fig. 1b). This suggests that niclosamide may inhibit the growth of other fungal pathogens and thereby protect plants from fungal diseases. Benzothiadiazole and probenazole induce SA pathway-mediated defense responses in plants by acting as chemical inducers^13^,^14^,^34^,^54^,^55^,^56^,^57^, leading to strong resistance against bacterial and fungal pathogens. Niclosamide induces the expression of both SA- and JA-responsive genes in rice (Fig. 6). This indicates that niclosamide may also be effective against fungi due to JA. Further study of the effects of niclosamide against other fungal pathogens will indicate whether the compound can protect plants from diseases caused by fungal pathogens.

The biochemical function and physiological action of niclosamide in human health and disease have been elucidated. Niclosamide inhibits glucose uptake, mitochondrial oxidative phosphorylation, and anaerobic metabolism in parasitic helminths^44^. It also inhibits transcription and DNA binding of the NFκΒ pathway, and increases ROS levels to induce apoptosis in acute myelogenous leukemia cells^50^. Recent work reports that niclosamide inhibits *Pseudomonas aeruginosa* quorum sensing^58^. These results suggest that niclosamide controls signaling networks of prokaryotes and eukaryotes by modulating metabolic and signaling pathways. Currently, we do not know the mechanism underlying niclosamide-mediated inhibition of *Xoo* growth and induction of SA-and JA-dependent plant defense responses. Future work will examine the effects of niclosamide on glucose metabolism, the transcriptome and proteome, and quorum sensing to identify the mechanism by which niclosamide inhibits bacterial leaf blight in rice. Further investigations of the effects of niclosamide on mitochondrial oxidative phosphorylation, changes in transcriptome and ROS levels, and changes in hormone signals (including SA and JA) also are required to fully characterize its functions as a broad-spectrum signaling regulator in plants.

The use of antibiotics and chemicals in plant agriculture is a subject of some concern. The use of antibiotics and chemicals in open fields over large expanses of land may increase the frequency of antibiotic resistance in gene pools, or possible chemical accumulation in plant tissues and the food chain. Niclosamide is a teniacide, which is effective against cestodes such as tapeworms that infect humans and many other animals (although it is not effective against as pinworms and roundworms). Niclosamide also is used as a piscicide. Recent work reported that it appeared to be safe and well tolerated in humans, even when used in mass treatment campaigns in several countries, although it has known adverse effects including nausea, retching, abdominal pain, light headedness, pruritus, vomiting, and dizziness^59^. There are no previous reports of niclosamide effects on plant growth, other organisms, or the food chain. We cannot state that niclosamide treatment of agricultural crops is safe for other organisms or for the food chain because research in this area is still lacking. Therefore, further studies on the function and stability of niclosamide under natural environmental conditions and when applied to crop plants are required to determine whether niclosamide has adverse effects on other organisms and human health when applied to plants.

In conclusion, the results presented herein indicate that niclosamide protects rice plants from bacterial leaf blight by inhibiting *Xoo* growth, inducing SA accumulation, and/or by inducing the expression of defense-related gene pathways. Further functional studies on niclosamide-mediated growth inhibition of bacterial and fungal pathogens, and elucidation of its role in plant signaling pathways, will provide information about the mechanism underlying niclosamide activity in plants. Field tests evaluating the effects of niclosamide application on rice plants will provide information on whether it has adverse effects on other organisms or the food chain.

## Methods

### Plant materials and chemicals

The rice (*Oryza sativa*) japonica cultivar, Nipponbare, was used in this study. Before treatment with the pathogen or niclosamide, plants were grown in a greenhouse maintained at 31 °C in the light and 25 °C in the dark under a 16 h photoperiod until the booting stage. The chamber was equipped with incandescent lights. Stock solutions of niclosamide (Sigma-Aldrich) and parthenolide (Sigma-Aldrich) were made by dissolving the chemicals in dimethyl sulfoxide (DMSO) at a concentration of 5 mg/ml. The stock solutions were diluted in distilled water before use.

### Examining the effect of niclosamide on *Xoo* bacterial growth

The effect of niclosamide on the growth of *Xoo* strains PXO99, 10208, and K3a, and a *Xag* strain, and the MIC of niclosamide against the PXO99 and 10208 strains were investigated. *Xoo* and *Xag* strains were obtained from the Rural Development Administration, Korea. Bacterial strains were cultivated in peptone-sucrose broth containing 15 μg/ml of the antibiotic cephalexin (Sigma-Aldrich), and grown to an optical density of 1.0 at 600 nm. Then, 2 μl of each culture was grown on agar medium containing different concentrations of niclosamide (0−50 μg/ml) to examine the effects of niclosamide on bacterial growth. Inoculated plates were incubated at 28 °C for 48 h and then photographed. The same method was used to determine whether parthenolide had bactericidal activity against the PXO99 and 10208 strains. The bactericidal activity of niclosamide on three different *E. coli* strains, Top10, Rosseta2, and DH10b, also was examined. The *E. coli* strains were cultivated in LB medium to an optical density of 1.0 at 600 nm. Then, 2 μl of each culture was grown on agar medium containing different concentrations of niclosamide. Inoculated plates were incubated at 37 °C for 24 h and photographed.

To determine the MIC, 2 μl of each culture were inoculated onto peptone-sucrose agar (PSA) medium containing 0−5.0 μg/ml niclosamide. Bacterial growth was not detected at a concentration of 5.0 μg/ml niclosamide. Thus, to determine a more specific MIC value, the MIC of niclosamide against both *Xoo* strains was narrowed down from 4.0 to 4.8 μg/ml. The lowest concentration of niclosamide (4.2 μg/ml) with no visible growth was taken as the MIC.

To examine the effects of niclosamide on fungal growth, 12 *M. oryzae* strains (46531, 46532, 46534, 46535, 46536, 46538, 46540, 46541, 46542, 46544, KI215, and PO6-6) were inoculated onto potato dextrose agar (Difco) medium containing different concentrations of niclosamide (0−50 μg/ml). The plates were then incubated at 28 °C for 72 h and photographed.

### Effect of niclosamide dosage on rice disease responses

To determine the minimum amount of niclosamide needed to inhibit the development of leaf blight in rice, the rice cultivar Nipponbare and *Xoo* strain PXO99 were used. PXO99 cells were prepared as follows. A single PXO99 colony was suspended in peptone-sucrose broth and plated onto fresh PSA medium. After 2 days of culture at 28 °C, the cells were collected and centrifuged to remove exopolysaccharides. The cells were then resuspended in double-distilled water, producing more than 10^9^ cell forming units (cfu) per ml.

The dosage effect of niclosamide on PXO99 lesion development was evaluated by measuring the lesion length (cm) and bacterial growth in the leaves of niclosamide-treated rice. To treat rice with PXO99, plants were grown for 80 days in a greenhouse at 31 °C in the light and 25 °C in the dark under a 16 h photoperiod until the booting stage. The greenhouse humidity was maintained over 90%. The fully expanded uppermost leaves were inoculated with PXO99 by the leaf-clipping method, followed by treatment with various doses (0, 4, 8, 20, and 40 μg/ml) of niclosamide applied by foliar spraying every 4 days. As a control, mock inoculation was conducted with distilled water. After 16 days, the leaves were photographed and lesion length was measured using ImageJ Software. All data are expressed as the mean values from 15 leaves per treatment. The experiment was repeated five times under the same conditions. To estimate bacterial growth, leaves were cut off the plants and ground with a mortar and pestle. After grinding, each sample was suspended in sterile water and plated onto PSA medium containing 15 μg/ml cephalexin. The bacterial population was scored by counting the number of colonies every 4 days after inoculation. The lesion lengths and bacterial populations were expressed as the mean value plus/minus the standard deviation.

### Niclosamide effects on disease responses in rice

Rice plants were inoculated with *Xoo* and sprayed with niclosamide. PXO99 cells and plants were prepared as described above. To treat rice with PXO99, the fully expanded uppermost leaves of 80-day-old rice plants were inoculated with PXO99 by the leaf-clipping method. The samples were incubated for 4 days until leaf blight lesions with a length of 2 cm developed, after which the leaves were treated with 8 μg/ml niclosamide by foliar spraying every 4 days. As a control, mock inoculation was conducted with distilled water. Leaves were photographed after 16 days, and lesion length and bacterial growth estimated as described above. All data are expressed as the mean values of 15 leaves per treatment. The experiment was repeated five times under the same conditions. The lesion length and bacterial population were expressed as the mean value plus/minus the standard deviation.

### Systemic effect of niclosamide on rice disease responses

To examine the systemic effect of niclosamide on *Xoo*-mediated leaf blight development, the fully expanded uppermost leaves of 80-day-old rice plants were inoculated with PXO99 by the leaf-clipping as described above. Half of the leaves were completely covered with polythene bags and half were sprayed with 8 μg/ml niclosamide. The local leaves were then sprayed with 8 μg/ml niclosamide every 4 days. Both local and systemic leaves were photographed after 16 days. Lesion length and bacterial growth were estimated as described above. The experiment was repeated five times under the same conditions. The lesion length and bacterial population were expressed as the mean value plus/minus the standard deviation.

### Quantification of systemically translocated niclosamide

Half of the leaves of 80-day-old rice plants were covered by polythene bags and the remaining systemic leaves were sprayed with 8 μg/ml niclosamide. After treatment, the bag-covered leaves were harvested after incubation for the indicated time periods. Niclosamide was extracted from 0.5 g of each sample using absolute MeOH, and the niclosamide concentration was determined by HPLC separation and fluorescence detection. The relative niclosamide concentration in each sample was compared with the 100 ppb niclosamide standard. Niclosamide levels were expressed as the mean value plus/minus the standard deviation.

### Determination of salicylic acid (SA) and its conjugate

SA and the SA conjugate glucosyl-SA were extracted from 0.5 g of rice leaves that were treated with 8 μg/ml niclosamide for 4 h. Samples were collected at five time points (0, 0.5, 1, 2, and 4 h). The concentrations of SA and glucosyl-SA were measured by HPLC as described previously^60^.

### Estimation of pathogen-related gene transcript levels in niclosamide-treated rice

The rice cultivar Nipponbare was sprayed with 40 μg/ml niclosamide as described above. Total RNA was extracted from niclosamide-treated and untreated leaves at 0, 0.5, 12, and 24 h, quantified, and diluted to equal concentrations. All niclosamide-treated or untreated plants were used for total RNA extraction. First-strand cDNA was synthesized using 1 μg of total RNA and an iScript cDNA Synthesis Kit (Bio-Rad). An equal volume of cDNA was amplified by quantitative real-time RT-PCR (MyiQ, Bio-Rad) according to the manufacturer’s protocol. Then, 10 nM of specific primers and template cDNA were combined with 25 μl iQ SYBR Green Super Mix (Bio-Rad) and the amplification reaction performed under the following thermal cycling conditions: 95 °C for 10 min; 45 cycles of 95 °C for 10 s; 60 °C for 10 s; and 72 °C for 10 s. The CT values of target genes were normalized to the CT value of the *actin1* gene and analyzed with iCycler IQ software (Bio-Rad). The experiments were repeated three times. PCR primers were designed using Primer3Plus (http://www.bioinformatics.nl/cgi-bin/primer3plus/primer3plus.cgi/). Primer specificity was verified by cloning into the pGEM T-Easy vector (Promega) and sequencing with an ABI 3730 × l DNA Analyzer (Applied Biosystems). The primers used for quantitative PCR were as follows: *OsPR1* forward, 5′-TTATCCTGCTGCTTGCTGGT-3′; *OsPR1* reverse, 5′-GATGTTCTCGCCGTACTTCC-3′; *OsPR10* forward, 5′-GGCACCATCTACACCATGAA-3′ and *OsPR10* reverse, 5′-TTGTCGGCTGTGATGAATGT-3′; *OsWRKY45* forward, 5′-CCGGCATGGAGTTCTTCAAG-3′ and *OsWRKY45* reverse, 5′-TATTTCTGTACACACGCGTGGAA-3′; *OsWRKY62* forward, 5′-AGGATGGGTACCAATGGA-3′ and *OsWRKY62* reverse, 5′-ACGAGTTGATGGAGATGGA-3′; *OsWRKY71* forward, 5′-AGCCCAAGATCTCCAAGCTC-3′ and *OsWRKY71* reverse, 5′-ACGAGGATCGTGTTGTCCTC-3′; *OsHI-LOX* forward, 5′-GCATCCCCAACAGCACATC-3′ and *OsHI-LOX* reverse, 5′-AATAAAGATTTGGGAGTGACATATTGG-3′; *OsACS2* forward, 5′-GGAATAAAGCTGCTGCCGAT-3′ and *OsACS2* reverse, 5′-TGAGCCTGAAGTCGTTGAAGC-3′.

### Niclosamide effect on rice growth and development

Three-week-old Nipponbare leaves were sprayed with 8 μg/ml niclosamide at 4-day intervals until seed maturation. The phenotypic characteristics of untreated and niclosamide-treated plants were measured at 70 days after planting. Grain phenotypes were examined after seed maturation.

## Additional Information

**How to cite this article**: Kim, S.-I. *et al.* Niclosamide inhibits leaf blight caused by *Xanthomonas oryzae* in rice. *Sci. Rep.* **6**, 21209; doi: 10.1038/srep21209 (2016).
